# Rare Fungal Illness Follows Tornado

**DOI:** 10.1289/ehp.121-a116

**Published:** 2013-04-01

**Authors:** Bob Weinhold

**Affiliations:** Bob Weinhold, MA, has covered environmental health issues for numerous outlets since 1996. He is a member of the Society of Environmental Journalists.

More than 1,000 people were injured when a severe tornado ripped through Joplin, Missouri, on 22 May 2011, and 158 eventually died.^1^ Within a few days of the tornado, several of the injured began to suffer from a fungal infection suspected to be cutaneous necrotizing mucormycosis. Doctors scrambled to do what they could, but testing to identify the specific causal fungus lagged, treatment (including surgery and medications) was sometimes ineffective, and five people died.^2^ A team of U.S. researchers who investigated the outbreak now report what they say is the largest known cluster of the disease, with 13 identified patients.^2^^,^^3^

The term “flesh-eating disease” is sometimes used to describe one of the obvious effects of diseases like mucormycosis, but this illness can also seriously affect many other body systems—including the pulmonary, sinus, rhinocerebral, gastrointestinal, cutaneous, and other body systems.^4^ In addition, many microbes other than the 10 fungi genera linked to mucormycosis can cause a so-called flesh-eating effect.^5^

The Joplin cases were caused by the fungus *Apophysomyces trapeziformis*, which is commonly found in soils, decaying vegetation, and water containing organic matter such as leaves and soil.^2^^,^^3^ *A. trapeziformis* and related species thrive in iron-rich, acidic environments and are known to particularly affect individuals with underlying diabetes mellitus, hematological malignancy, iron overload, or acidemia (abnormal blood acidity).^6^ These fungi have also been associated with a compromised immune system, malnourishment, transplant receipt, and prolonged corticosteroid use.^4^

When comparing the 13 Joplin cases with controls who were injured during the tornado but showed no evidence of mucormycosis, the researchers discovered a significant link between fungal infection and the occurrence of penetrating wounds (especially multiple wounds) containing wood, soil, gravel, and other foreign bodies. All 13 patients were injured in the heart of the path of the EF-5 tornado, which had winds over 200 mph.

There was no link with potential confounders such as age, sex, race, preexisting medical condition, or location of the wound(s).^2^ There also was no link with contaminated medical equipment, which has been implicated in some previous mucormycosis cases.^7^

The global incidence of mucormycosis cases is low, though other cases may have gone undetected. Three fungal genera, *Rhizopus*, *Mucor*, and *Lichtheimia*, account for most of the roughly 1,500 documented cases worldwide.^4^^,^^8^ Seven other genera, including *Apophysomyces*, account in total for about 200 or so known cases,^8^ and these cases are commonly linked with trauma from an accident, tornado, tsunami, volcanic eruption, burn, insect sting, or spider bite.^4^^,^^8^ Mortality rates range from about 35% to 100% depending on the presence of certain underlying conditions.^4^^,^^8^

**Figure:**
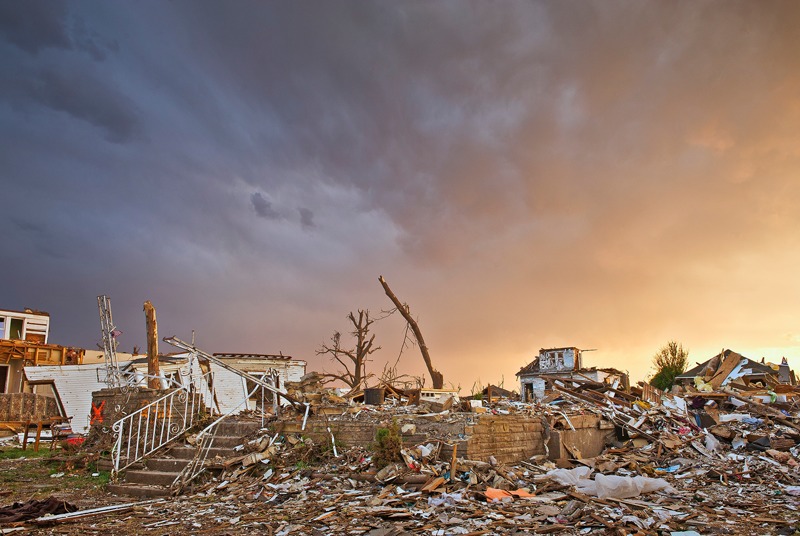
A Joplin, Missouri neighborhood lies in ruins after a mile-wide tornado tore through on 22 May 2011. © Jim Reed / Corbis

The increasing number of documented cases in recent decades leads some experts to consider mucormycosis an emerging disease.^2^^,^^4^ The observed link to extreme weather events suggests incidence could continue to rise if such events become more common with climate change, as expected.^9^ Another plausible factor is the expansion of certain vulnerable populations, such as those with diabetes mellitus^10^ or those using corticosteroids;^11^ incidence of some of the diseases treated with these medications (such as allergies and asthma) is rising substantially.^12^

Because of the lethality of mucormycosis and its rapid onset, increased awareness by medical professionals of its possible occurrence and rapid initiation of treatment likely will be beneficial, says Benjamin Park, a coauthor of two Joplin aftermath studies and epidemiology team leader of the U.S. Centers for Disease Control and Prevention’s Mycotic Diseases Branch. That rapid targeted treatment didn’t occur in Joplin, where the time from injury to first positive culture ranged from 6 to 24 days.^2^

Once doctors think to look for this kind of health problem, identification of these infections typically takes 3–5 days and is based on culture and/or histopathology, says Dimitrios Kontoyiannis, a professor of infectious diseases at the University of Texas M.D. Anderson Cancer Center, who wasn’t involved with the public health response to the Joplin outbreak. In other research, rapid treatment with amphotericin B or posaconazole led to the best outcomes.^13^ In Joplin, the 13 victims, including those who died, were treated with a range of antifungal medications individually or in combination, among them these two drugs.^2^

Craig Smith, medical director for infectious diseases at University Hospital in Augusta, Georgia, and a member of the Infectious Diseases Society of America’s Rapid Communications Work Group, says every medical student learns about fungal infections, but since such infections are rarely seen in most practices, they tend to drop out of the top tiers of possibilities considered for a patient. Smith knows every health-care professional can’t be an expert on mycoses. So he suggests that clinicians be particularly alert to patients with penetrating wounds or crush injuries accompanied by possible contamination with moist organic matter. They should suspect a possible fungal infection if the wound has an unusual feel, appearance, or drainage in the first day or two. “If a wound looks funny,” Smith says, “call an expert immediately.”
